# Prognostic value of preoperative circulating tumor cells for hepatocellular carcinoma with portal vein tumor thrombosis: A propensity score analysis

**DOI:** 10.1007/s00432-023-04834-8

**Published:** 2023-05-09

**Authors:** Jing-jing Yu, Ya-ni Li, Chang Shu, Hui-yuan Yang, Zhao Huang, Ran Tao, Yue-yue Chen, Xiao-ping Chen, Wei Xiao

**Affiliations:** Hepatic Surgery Center, Hubei Clinical Medicine Research Center of Hepatic Surgery, Hubei Key Laboratory of Hepato-Biliary-Pancreatic Diseases, Tongji Hospital, Tongji Medical College, Huazhong University of Science and Technology, Wuhan, 430030 China

**Keywords:** Circulating tumor cells, Hepatocellular carcinoma, Portal vein tumor thrombosis, Prognosis, Propensity score matching analysis

## Abstract

**Purpose:**

The role of circulating tumor cells (CTCs) in hepatocellular carcinoma (HCC) with portal vein tumor thrombosis (PVTT) is not fully understood.

**Methods:**

In this retrospective analysis, we included 316 HCC patients who underwent hepatectomy and preoperative CTC detection. We selected 41 pairs of matched HCC patients with and without PVTT using propensity score matching (PSM) analysis. We compared the preoperative CTC counts in patients from both the full cohort and the PSM model. We also analyzed their associations with disease-free survival (DFS) and overall survival (OS).

**Results:**

Before and after PSM analysis, the preoperative CTC counts in the HCC with PVTT group were substantially higher than in the HCC without PVTT group. In both the full cohort of patients and the PSM model, patients with CTC ≥ 2 had significantly shorter OS and DFS than patients with CTC < 2. The outcomes of HCC patients with PVTT could be well differentiated by preoperative CTC levels. HCC patients with CTC ≥ 2 had noticeably shorter OS (9.9 months vs. 24.6 months, P = 0.0003) and DFS (6.0 months vs. 12.3 months, P = 0.0041) than those with CTC < 2. Moreover, preoperative CTC ≥ 2 remained an independent predictor in all groups’ multivariate analysis.

**Conclusion:**

We discovered a link between preoperative CTC counts and the occurrence of PVTT and confirmed the prognostic significance of preoperative CTC in HCC patients with PVTT. These findings suggest that preoperative CTC counts have the potential to assist in identifying patients with HCC and PVTT who may benefit from surgery.

**Supplementary Information:**

The online version contains supplementary material available at 10.1007/s00432-023-04834-8.

## Introduction

Liver cancer is the sixth most prevalent cancer globally (Siegel 2021). About 90% of all liver cancers are hepatocellular carcinomas (HCC), with China accounting for nearly half of all new cases and deaths each year (Cao 2021). HCC generally has a dismal prognosis, with a 5-year overall survival rate of only 10–15% (EASL 2012; Omata 2017; Marrero 2018). This is mainly because 70–80% of patients are diagnosed at advanced stages when there are no obvious early symptoms (Cheng et al. 2020).

HCC cells are predisposed to spreading via the portal vein branches and forming portal vein tumor thrombosis (PVTT) due to the biological properties of HCC and the anatomical features of the liver (Cheng et al. 2020). According to surgical resection studies, the prevalence of PVTT is approximately 40% (Sakamoto and Nagano 2017). PVTT generally has an extremely poor prognosis, with a median survival time of only two to four months when treated with best supportive care (Schoniger-Hekele 2001; Liu 2018).

Currently, there is a lack of global consensus or guidelines for the management of HCC with PVTT. In Europe and America, according to the Barcelona Clinic Liver Cancer Staging (BCLC) guidelines, HCC with PVTT is classified as BCLC Stage C and should be treated with targeted drugs such as Sorafenib and Lenvatinib (EASL 2018). However, in many Asian countries, including China, specialists argue that multidisciplinary therapy, including surgery, transcatheter arterial chemoembolization (TACE), radiotherapy (RT), and targeted therapy should be considered to improve treatment outcomes (Cheng et al. 2020). In particular, for some selected HCC patients with PVTT, liver resection coupled with thrombectomy has emerged as a relatively curative therapeutic option due to recent developments in surgical procedures and perioperative care (Zhang 2019, Cheng et al. 2020). Unfortunately, only a small number of carefully selected individuals can undergo such curative surgery. As a result, it is important to identify patients who may benefit from surgical therapy and have a better prognosis (Qiu 2021).

Circulating tumor cells (CTCs) are tumor cells that enter the peripheral blood circulation either on their own or as a result of diagnostic and therapeutic intervention (Hosseini 2016). As the origin of cancer metastasis, CTCs have been verified as a diagnostic and prognostic biomarker for many malignancies. They serve in the in vitro early tumor diagnosis, prognosis, and survival prediction, monitoring of drug resistance, detecting recurrence, and evaluating drug efficacy to support the decision-making process for treatments and the modification of treatment regimens (Cristofanilli 2004, Cohen 2008, de Bono 2008). Our previous study showed that perioperative CTC counts could predict the prognosis of HCC patients undergoing hepatectomy (Yu 2018, 2021). However, the role of CTCs in HCC with PVTT is not fully understood. In this study, we evaluated the relationship between preoperative CTC counts and the presence of PVTT in patients with HCC who underwent hepatic resection. We also evaluated the prognostic significance of preoperative CTC levels in HCC patients, particularly in those with combined PVTT. Our findings suggest that preoperative CTC counts have the potential to assist in identifying patients with HCC and PVTT who may benefit from surgical therapy and have a better prognosis.

## Methods

### Study design

**Fig. 1:**
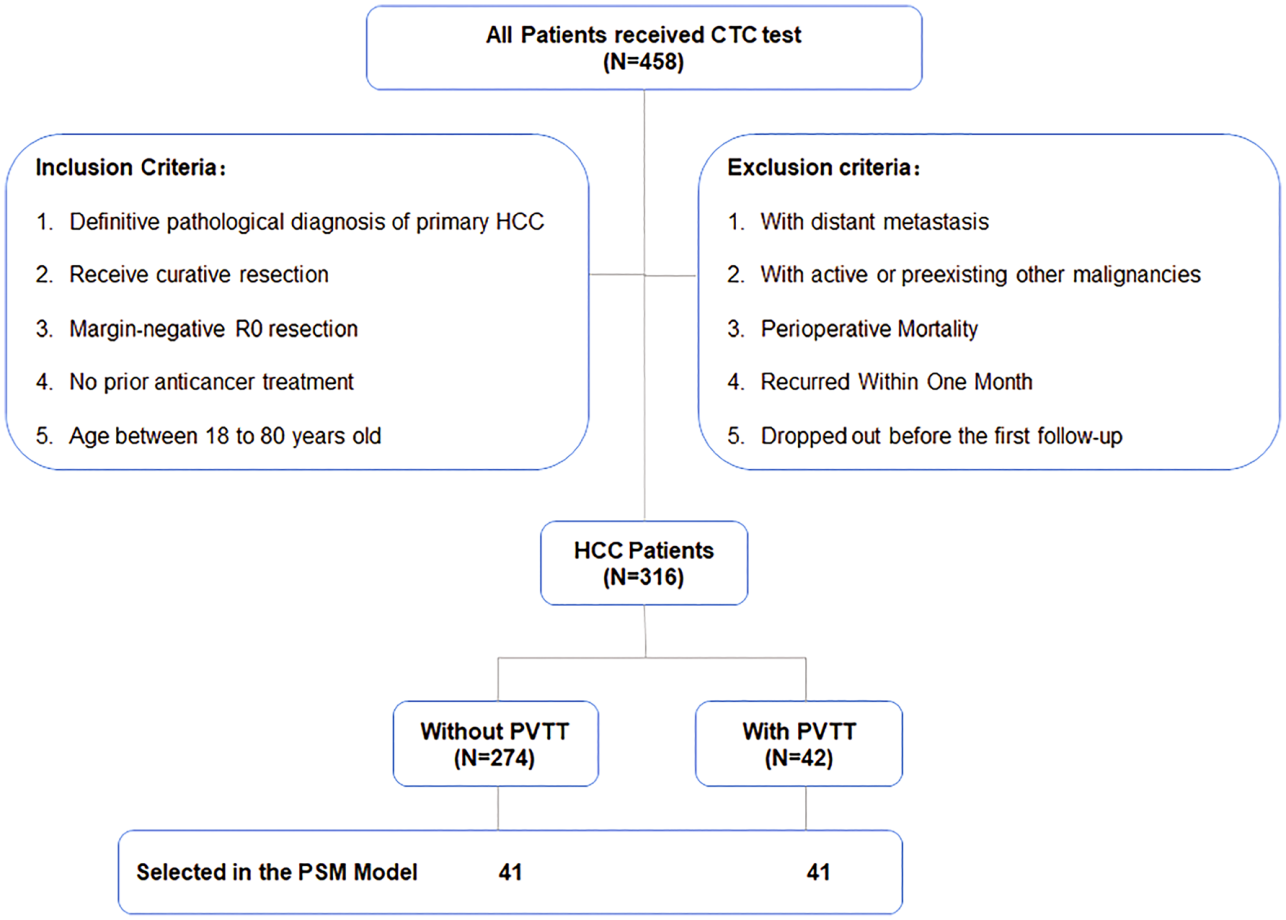
Patient distribution based on portal vein tumor thrombosis (PVTT) in the entire cohort. From 458 patients received CTC detection, 316 patients were included in this study based on certain inclusion and exclusion criteria. 42 (13.3%) of them have PVTT, compared to 274 (86.7%) of the patients who do not have it. For further analysis, 41 pairs of matched patients were selected using the propensity score matching (PSM) model

As summarized in Fig. 1, between December 2013 and August 2015, 458 patients received CTC detection at the Hepatic Surgery Center, Tongji Hospital, Tongji Medical College, Huazhong University of Science and Technology. Of these, 316 patients were included in this retrospective study. The inclusion criteria were:(1) definitive pathological diagnosis of primary HCC; (2) receipt of curative resection; (3) margin-negative R0 resection; (4) no prior anticancer treatment; and (5) age between 18 and 80 years. The exclusion criteria were: (1) presence of distant metastasis; (2) active or preexisting other malignancies; (3) perioperative mortality; (4) recurrence within one month; and (5) dropout before the first follow-up. The same surgical and oncological principles were followed in this department. A five-year follow-up was conducted periodically by phone calls and counterchecks. We reanalyzed the detection and clinical data in this study. The study was approved by the ethics committee of Tongji Hospital and all patients provided informed consent.

### CTC analysis

One day before surgery, preoperative peripheral blood samples were collected. Blood sampling, CTC detection processing, and data analysis with the CellSearch System have been previously reported (Yu 2018, 2021). Intact cells stained with positive DAPI and cytokeratin, but negative CD45 are defined as CTCs. Two trained researchers independently interpreted the visual images.

### Classification of PVTT

Cheng’s classification was used to classify the extent of PVTT in patients with HCC (Cheng et al. 2020, Sun et al. 2022). PVTT was classified into four types based on the extent of tumor thrombus in the portal vein: Type I: tumor thrombus involving segmental or sectoral branches of the portal vein or above; Type II: tumor thrombus involving the right or left portal vein; Type III: tumor thrombus involving the main portal vein; and Type IV: tumor thrombus involving the superior mesenteric vein.

### Propensity score matching (PSM) analysis

To reduce potential confounding effects and treatment selection bias, we conducted a propensity score matching analysis using the “MatchIt” package in R (version 3.6.3) (Zhang 2017). We comprehensively selected possible variables associated with PVTT and outcomes of HCC for propensity score generation, including age (≤ 50/ > 50 years), gender (male/female), HBsAg (negative/positive), liver cirrhosis (yes/no), Child–Pugh score (A/B), largest tumor size (≤ 5/ > 5 cm), tumor number (single/multiple), Edmonson grade (I-II/III-IV), and AFP (≤ 400/ > 400 ng/ml). Then, we calculated a 1:1 match between the HCC with PVTT group and the HCC without PVTT group using nearest neighbor matching with a caliper width of 0.02.

### Statistical analysis

According to our previous studies (Yu 2018, 2021), we tested whether a preoperative CTC count of 2 was a proper cutoff value for this study. As it could clearly distinguish patients with longer OS from those with shorter OS in each subgroup of the cohort, we used it as the cutoff value in this study. We used Fisher’s exact test and chi-squared tests to determine the proportional differences between groups. We used the Wilcoxon matched-pairs signed rank test and Mann–Whitney test to compare differences in CTC counts between the HCC with PVTT group and the HCC without PVTT group in all patients and matched pairs of patients, respectively. We used Kaplan–Meier and log-rank tests to estimate and compare patients’ outcomes of overall survival (OS) and disease-free survival (DFS). We used univariate and multivariate Cox proportional regression analysis to examine OS- and DFS-related factors. All statistical analyses were performed using SPSS (version 21.0), and P < 0.05 was considered statistically significant.

## Results

### Characteristics of patients

As shown in Fig. 1, 316 patients with HCC undergoing curative liver resection were enrolled in this retrospective study. Of these, 42 patients were diagnosed with PVTT and 41 matched pairs of patients with and without PVTT were identified and included in the PSM analysis. Table 1 compares the baseline demographics of patients before and after PSM analysis. Prior to matching, the two groups did not differ significantly in terms of age, gender, HBsAg, liver cirrhosis, Child–Pugh score, or tumor number. Patients with PVTT had increased tumor size (P = 0.000), higher Edmonson grade (P = 0.030), higher serum AFP levels (P = 0.004), and consequently higher BCLC stage (P = 0.000). After matching, all these differences were balanced out (P > 0.05).

**Table 1:** Clinical characteristics of HCC patients before and after propensity score matching analysis

Clinical characteristics	All Patients			Patients in PSM Model		
	Without PVTT (N = 274)	With PVTT (N = 42)	P	Without PVTT (N = 41)	With PVTT (N = 41)	P
Age, years			0.618			1.000
≤ 50	156	22		22	22	
> 50	118	20		19	19	
Gender			1.000			1.000
Male	239	37		35	35	
Female	35	5		6	6	
HBsAg			0.835			1.000
Negative	53	9		9	8	
Positive	221	33		32	33	
Liver cirrhosis			1.000			1.000
No	76	12		11	11	
Yes	198	30		30	30	
Child–Pugh score			0.175			1.000
A	258	37		36	36	
B	16	5		5	5	
No. of tumor			0.176			1.000
Single	211	28		28	28	
Multiple	63	14		13	13	
Largest tumor size,			0.000			1.000
≤ 5 cm	122	4		4	4	
> 5 cm	152	38		37	37	
Edmondson grade			0.030			1.000
I-II	167	18		19	18	
III-IV	107	24		22	23	
AFP, ng/mL			0.004			1.000
Low (< 400)	177	17		16	17	
High (≥ 400)	97	25		25	24	
BCLC stage			0.000			0.494
0-A	107	0		2	0	
B + C	167	42		39	41	
Preoperative CTCs			0.000			0.000
< 2	225	14		35	14	
≥ 2	49	28		6	27	

### Association between PVTT and CTC

**Fig. 2:**
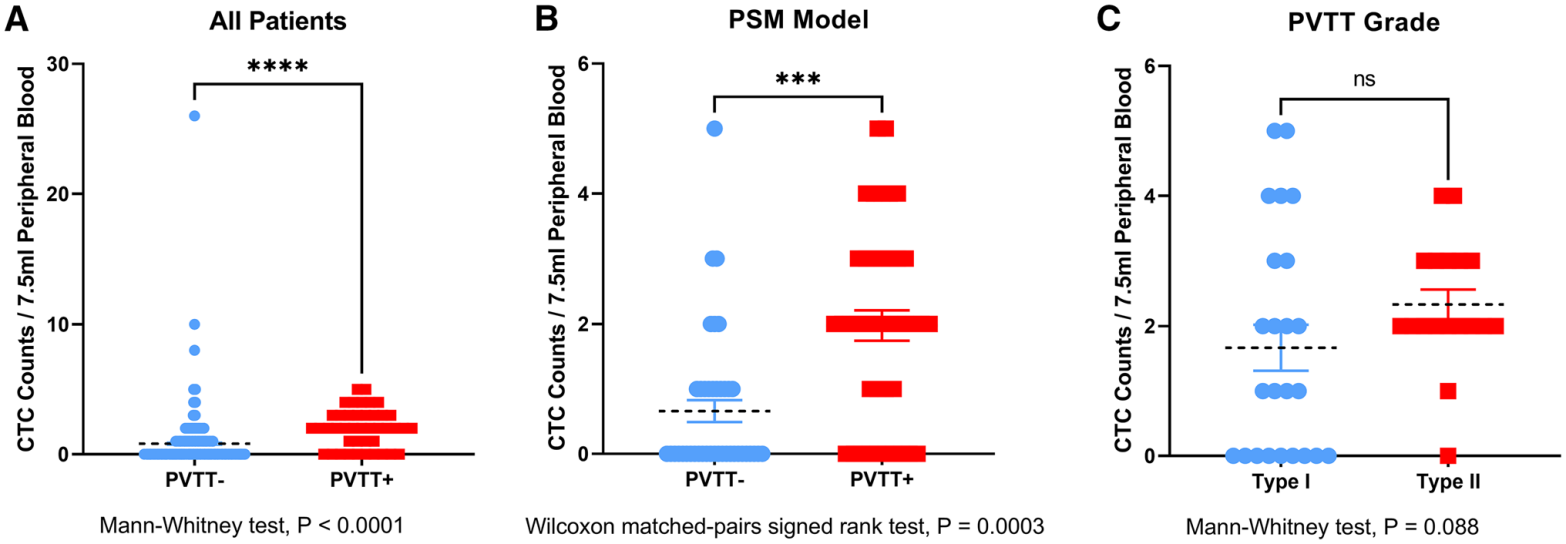
Comparison of preoperative CTC counts in different PVTT groups. **A** HCC patients without PVTT versus HCC patients with PVTT in the full cohort, Mann–Whitney test, P < 0.0001; **B** HCC patients without PVTT versus HCC patients with PVTT in the PSM model, Wilcoxon matched-pairs signed rank test, P = 0.0003; **C** Type I PVTT versus Type II PVTT (Cheng’s classification), Mann–Whitney test, P = 0.088. Asterisk for significant, ns for not significant

We observed a significant association between preoperative CTC counts and presence of PVTT in our full cohort. In HCC patients without PVTT, 49 out of 274 (17.9%) patients had ≥ 2 CTCs; while in HCC patients with PVTT, 28 out of 42 (66.7%) patients had ≥ 2 CTCs (Table 1, Chi-squared test, P = 0.000). The number of CTCs in the HCC with PVTT group was considerably higher than in the HCC without PVTT group (Fig. 2A, Mann–Whitney test, P < 0.0001). This significant association of PVTT with CTC persisted in the propensity score matching model: In HCC patients without PVTT, 6 out of 41 (14.6%) patients had ≥ 2 CTCs; while in HCC patients with PVTT, 27 out of 41 (65.9%) patients had ≥ 2 CTCs (Table 1, Chi-squared test, P = 0.000). The preoperative CTC counts remained higher in the HCC with PVTT group than in the HCC without PVTT group (Fig. 2B, Wilcoxon matched-pairs signed rank test, P = 0.0003). Moreover, we further compared the number of CTCs in different grades of PVTT; although the result did not meet the criteria for significance, there was a tendency towards an increased number of CTCs in type II PVTT (Cheng’s classification) compared to that in type I PVTT (Fig. 2C, Mann–Whitney test, P = 0.088). Interestingly, we also observed a significant association between preoperative CTC counts and presence of microvascular invasion (MVI) in our cohort: The number of CTCs in the HCC with MVI group was significantly higher than that in the HCC without MVI group (supplementary Fig. S1, Mann–Whitney test, P < 0.0001).

### Prognostic value of preoperative CTC in HCC

Table 2 compares the clinical characteristics of HCC patients between preoperative CTC ≥ 2 and < 2. In the full cohort of HCC patients, preoperative CTC ≥ 2 had marginally significant association with increased tumor number (P = 0.067) and higher Edmonson grade (P = 0.064), while it had highly significant association with increased tumor size (P = 0.000), presence of PVTT (P = 0.000), and consequently higher BCLC stage (P = 0.000). In HCC patients with PVTT, those with preoperative CTC ≥ 2 had significantly increased tumor size (P = 0.012). Since HCC patients with PVTT were classified as BCLC stage C, the association of CTC with BCLC and PVTT was not statistically analyzed. However, it is still evident that the proportion of patients with preoperative CTC ≥ 2 is greatly increased.

**Table 2:** Comparison of clinical characteristics of HCC patients between preoperative CTC ≥ 2 and < 2

Clinical characteristics	All Patients			Patients with PVTT		
	CTC < 2 (N = 239)	CTC ≥ 2 (N = 77)	P	CTC < 2 (N = 15)	CTC ≥ 2 (N = 27)	P
Age, years			0.694			1.000
≤ 50	133	45		8	14	
> 50	106	32		7	13	
Gender			0.560			0.073
Male	207	69		15	21	
Female	32	8		0	6	
HBsAg			0.409			0.123
Negative	44	18		1	8	
Positive	195	59		14	19	
Liver cirrhosis			0.467			0.485
No	64	24		3	9	
Yes	175	53		12	18	
Child–Pugh score			0.607			0.639
A	224	71		14	23	
B	15	6		1	4	
No. of tumor			0.067			1.000
Single	187	52		10	18	
Multiple	52	25		5	9	
Largest tumor size,			0.000			0.012
≤ 5 cm	117	9		4	0	
> 5 cm	122	68		11	27	
Edmondson grade			0.064			0.754
I-II	147	38		7	11	
III-IV	92	39		8	16	
AFP, ng/mL			0.107			0.744
Low (< 400)	153	41		7	10	
High (≥ 400)	86	36		8	17	
BCLC stage			0.000			/
0-A	101	6		0	0	
B + C	138	71		15	27	
PVTT			0.000			/
No	225	49		0	0	
Yes	14	28		15	27	

**Fig. 3:**
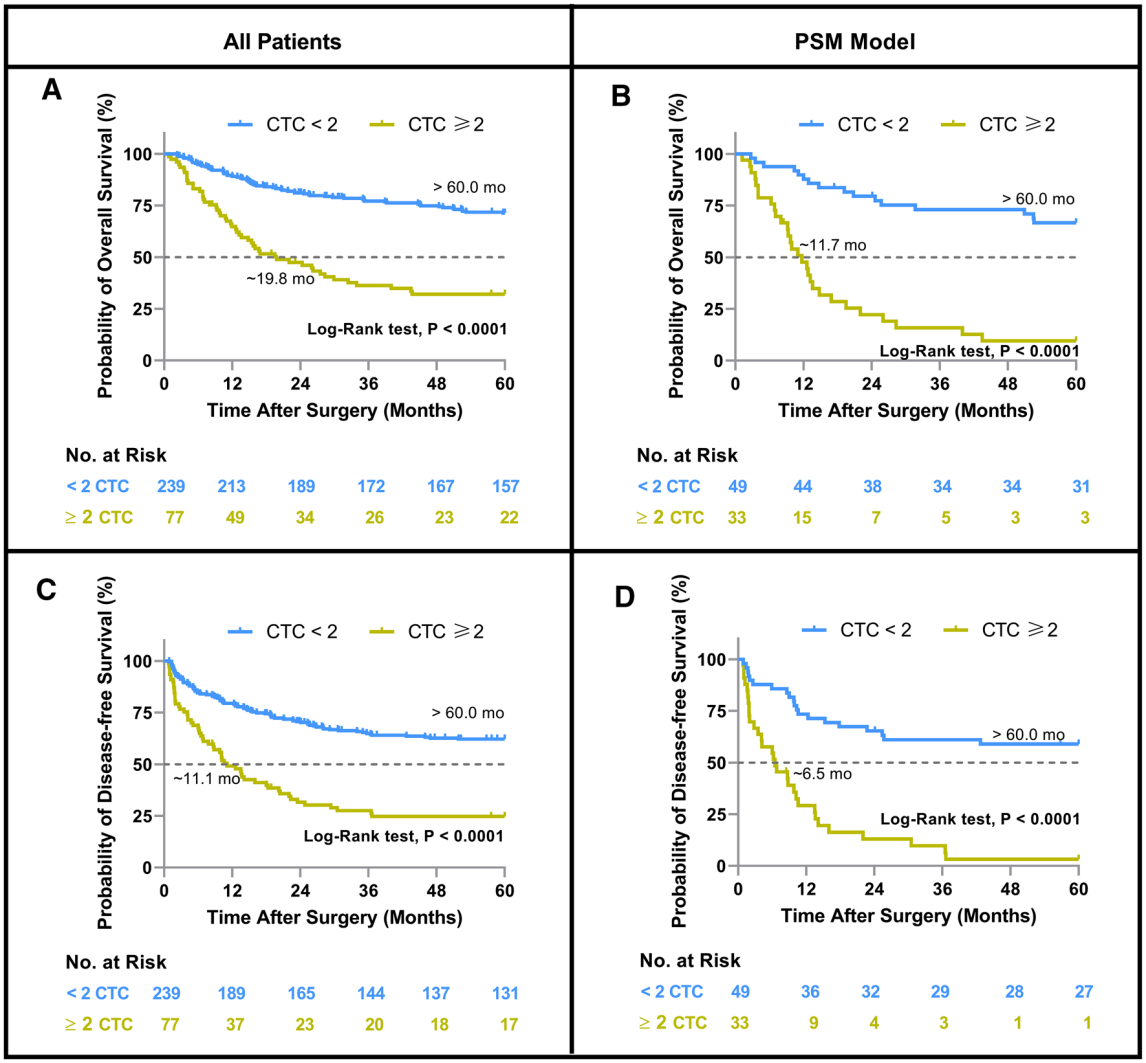
Probabilities of overall survival (Hosseini) and disease-free survival (DFS) in patients with HCC based on Kaplan–Meier analysis. **A** OS in the full cohort of patients (n = 316), preoperative CTC ≥ 2 *vs* CTC < 2; **B** OS in the PSM model (n = 82), preoperative CTC ≥ 2 *vs* CTC < 2; **C** DFS in the full cohort of patients (n = 316), preoperative CTC ≥ 2 *vs* CTC < 2; **D** DFS in the PSM model (n = 82), preoperative CTC ≥ 2 *vs* CTC < 2

The prognostic value of preoperative CTCs was evaluated in the full cohort of patients and in the PSM model using a cutoff value of 2 for preoperative CTC counts. In the full cohort of patients, patients with CTC ≥ 2 had significantly shorter OS (Fig. 3A, 19.8 months vs. not reached, P < 0.0001) and DFS (Fig. 3C, 11.1 months vs. not reached, *P* < 0.0001) than those with CTC < 2. Similarly, in the PSM model, patients with CTC ≥ 2 had significantly shorter OS (Fig. 3B, 11.7 months vs. not reached, P < 0.0001) and DFS (Fig. 3D, 6.7 months vs. not reached, P < 0.0001) than those with CTC < 2.

Univariate and multivariate Cox proportional regression analyses were subsequently performed. Due to the small number of significant factors in the univariate analysis of the propensity score matching (PSM) model, all clinical parameters were included in the multivariate analysis to ensure consistency in the analysis process, regardless of whether they were significant in the univariate analysis. (BCLC stage was excluded to avoid potential bias as it was a composite index consisting of tumor-related characteristics and liver function). As shown in Table 3, in the full cohort, higher Edmonson stage (P = 0.048), multiple tumor number (P = 0.000), increased tumor size (P = 0.000), higher serum AFP level (P = 0.000), higher BCLC stage (P = 0.000), presence of PVTT (P = 0.000), and preoperative CTC ≥ 2 (P = 0.000) were significantly related to patients’ OS in univariate analysis; meanwhile, multiple tumor number (P = 0.000), increased tumor size (P = 0.000), higher serum AFP level (P = 0.000), higher BCLC stage (P = 0.000), presence of PVTT (P = 0.000), and preoperative CTC ≥ 2 (P = 0.000) were also significantly correlated with patients’ DFS in univariate analysis. In the multivariate analysis of the full cohort of patients, largest tumor size (P = 0.000), presence of PVTT (P = 0.000), and preoperative CTC ≥ 2 (P = 0.001) remained significant and independent for patients’ OS; largest tumor size (P = 0.000), higher serum AFP level (P = 0.022), presence of PVTT (P = 0.000), and preoperative CTC ≥ 2 (P = 0.004) remained significant and independent for patients’ DFS.

**Table 3:** Univariate and multivariate Cox proportional regression analysis of factors associated with OS and DFS in all patients (N = 316)

Variables	Overall survival				Disease-free survival			
	Univariate analysis		Multivariate analysis		Univariate analysis		Multivariate analysis	
	HR (95% CI)	P	HR (95% CI)	P	HR (95% CI)	P	HR (95% CI)	P
Age, > 50 years *vs* ≤ 50 years	1.138 (0.792–1.634)	0.484			1.116 (0.806–1.544)	0.510		
Gender, male *vs* female	1.247 (0.746–2.085)	0.400			1.174 (0.732–1.882)	0.506		
HBsAg, positive *vs* negative	0.996 (0.636–1.559)	0.985			1.115 (0.738–1.683)	0.605		
Liver cirrhosis, yes *vs* no	0.795 (0. 539–1.173)	0.248			0.736 (0.519–1.043)	0.085		
Child–Pugh score, B *vs* A	1.072 (0.523–2.197)	0.850			0.941 (0.479–1.846)	0.859		
Edmonson stage, III-IV *vs* I-II	1.440 (1.004–2.066)	0.048	1.247 (0.860–1.809)	0.245	1.266 (0.914–1.754)	0.155		
No. of tumors, multiple *vs* single	2.181 (1.498–3.177)	0.000	1.191 (0.783–1.811)	0.415	2.217 (1.575–3.122)	0.000	1.393 (0.954–2.033)	0.086
Largest tumor size, > 5 *vs* ≤ 5 cm	4.969 (3.004–8.219)	0.000	3.147 (1.840–5.384)	0.000	3.677 (2.457–5.504)	0.000	2.471 (1.600–3.816)	0.000
AFP, ≥ 400 *vs* < 400 ng/mL	1.917 (1.335–2.752)	0.000	1.354 (0.903–2.032)	0.143	2.010 (1.451–2.784)	0.000	1.516 (1.061–2.165)	0.022
BCLC stage, B-C *vs* 0-A	5.675 (3.186–10.111)	0.000			3.737 (2.409–5.799)	0.000		
PVTT, yes *vs* no	5.650 (3.766–8.476)	0.000	3.078 (1.958–4.838)	0.000	4.326 (2.947–6.350)	0.000	2.622 (1.705–4.033)	0.000
Preoperative CTCs, ≥ 2 *vs* < 2	3.515 (2.435–5.074)	0.000	2.058 (1.364–3.105)	0.001	2.967 (2.121–4.151)	0.000	1.761 (1.196–2.592)	0.004

Table 4 shows the results of univariate and multivariate Cox proportional regression analyses performed for patients in the PSM model. After matching, only presence of PVTT (P = 0.000) and preoperative CTC ≥ 2 (P = 0.000) significantly correlated with patients’ OS and DFS in univariate analysis, and they remained significant and independent in the multivariate analysis.

**Table 4:** Univariate and multivariate Cox proportional regression analysis of factors associated with OS and DFS in patients selected in the PSM model (N = 82)

Variables	Overall survival				Disease-free survival			
	Univariate analysis		Multivariate analysis		Univariate analysis		Multivariate analysis	
	HR (95% CI)	P	HR (95% CI)	P	HR (95% CI)	P	HR (95% CI)	P
Age, > 50 years *vs* ≤ 50 years	1.422 (0.792–2.552)	0.238			1.122 (0.647–1.945)	0.682		
Gender, male *vs* female	1.647 (0.793–3.420)	0.181			1.372 (0.668–2.819)	0.389		
HBsAg, positive *vs* negative	0.578 (0.303–1.102)	0.096			0.767 (0.409–1.441)	0.411		
Liver cirrhosis, yes *vs* no	1.201 (0.608–2.371)	0.597			0.937 (0.506–1.734)	0.836		
Child–Pugh score, B *vs* A	0.684 (0.245–1.911)	0.469			0.542 (0.195–1.505)	0.240		
Edmonson stage, III-IV *vs* I-II	1.664 (0.910–3.041)	0.098			1.347 (0.771–2.353)	0.296		
No. of tumors, multiple *vs* single	1.530 (0.841–2.784)	0.164			1.687 (0.960–2.964)	0.069		
Largest tumor size, > 5 *vs* ≤ 5 cm	1.640 (0.508–5.294)	0.408			2.066 (0.643–6.641)	0.223		
AFP, ≥ 400 *vs* < 400 ng/mL	1.302 (0.712–2.380)	0.392			1.680 (0.945–2.988)	0.077		
BCLC stage, B-C *vs* 0-A	21.320 (0.020–22,572.489)	0.389			21.436 (0.037–12,308.272)	0.344		
PVTT, yes *vs* no	7.448 (3.581–15.490)	0.000	5.619 (2.413–13.087)	0.000	4.879 (2.610–9.119)	0.000	3.890 (1.814–8.342)	0.000
Preoperative CTCs, ≥ 2 *vs* < 2	5.940 (3.152–11.197)	0.000	3.737 (1.660–8.412)	0.001	4.974 (2.757–8.973)	0.000	2.465 (1.139–5.334)	0.022

### Prognostic value of preoperative CTC in PVTT

**Fig. 4:**
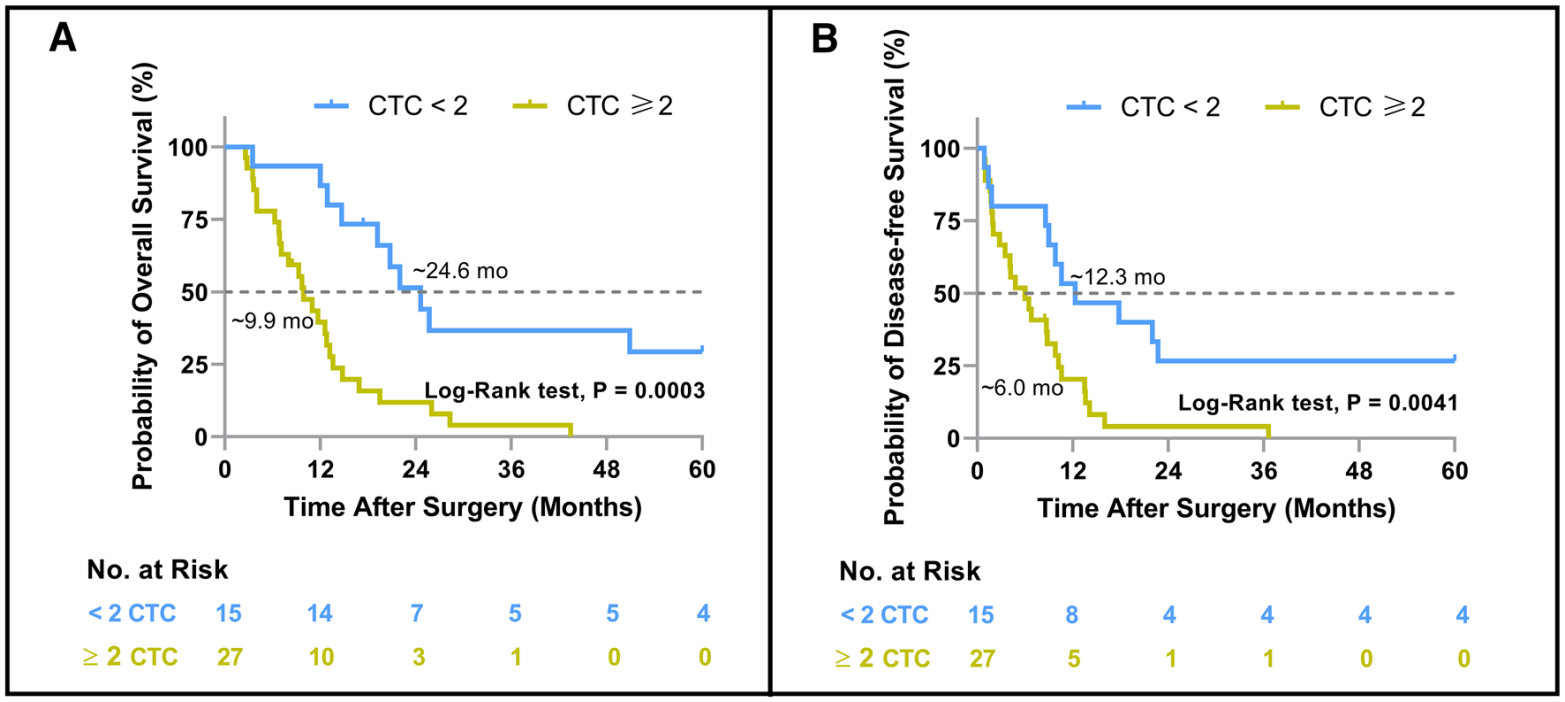
Probabilities of overall survival (Hosseini) and disease-free survival (DFS) in HCC patients with PVTT (n = 42) based on Kaplan–Meier analysis. **A** OS, preoperative CTC ≥ 2 *vs* CTC < 2; **B** DFS, preoperative CTC ≥ 2 *vs* CTC < 2

Finally, the prognostic value of preoperative CTCs was evaluated specifically in HCC patients with PVTT. The study found that the outcome of HCC patients with PVTT could be well differentiated by preoperative CTC levels. Patients with CTC ≥ 2 had significantly shorter OS (Fig. 4A, 9.9 months vs. 24.6 months, P = 0.0003) and DFS (Fig. 4B, 6.0 months vs. 12.3 months, P = 0.0041) than those with CTC < 2. Table 5 presents the results of univariate and multivariate Cox proportional regression analyses conducted for patients with PVTT. For OS, preoperative CTC ≥ 2 was significantly related to patients’ OS in univariate analysis (P = 0.001), and remained significant and independent in the multivariate analysis (P = 0.001). For DFS, higher serum alpha-fetoprotein (AFP) level (P = 0.029) and preoperative CTC ≥ 2 (P = 0.006) were significantly associated with patients’ DFS in univariate analysis, and in the multivariate analysis, they both remained significant and independent (AFP: P = 0.024; CTC: P = 0.045).

**Table 5:** Univariate and multivariate Cox proportional regression analysis of factors associated with OS and DFS in patients with PVTT (N = 42)

Variables	Overall survival				Disease-free survival			
	Univariate analysis		Multivariate analysis		Univariate analysis		Multivariate analysis	
	HR (95% CI)	P	HR (95% CI)	P	HR (95% CI)	P	HR (95% CI)	P
Age, > 50 years *vs* ≤ 50 years	1.144 (0.593–2.208)	0.687			1.058 (0.553–2.024)	0.864		
Gender, male *vs* female	1.854 (0.763–4.504)	0.173			1.841 (0.757–4.473)	0.178		
HBsAg, positive *vs* negative	0.932 (0.447–1.945)	0.775			1.063 (0.485–2.331)	0.878		
Liver cirrhosis, yes *vs* no	0.751 (0. 360–1.566)	0.852			0.847 (0.416–1.727)	0.649		
Child–Pugh score, B *vs* A	1.298 (0.453–3.715)	0.627			0.972 (0.343–2.753)	0.958		
Edmonson stage, III-IV *vs* I-II	1.784 (0.909–3.502)	0.092			1.407 (0.728–2.718)	0.310		
No. of tumors, multiple *vs* single	0.981 (0.497–1.936)	0.955			1.331 (0.679–2.611)	0.405		
Largest tumor size, > 5 *vs* ≤ 5 cm	2.992 (0.708–12.646)	0.136			3.296 (0.783–13.876)	0.104		
AFP, ≥ 400 *vs* < 400	1.424 (0.718–2.826)	0.312			2.154 (1.082–4.291)	0.029	2.846 (1.149–7.053)	0.024
Preoperative CTCs, ≥ 2 *vs* < 2	3.932 (1.798–8.597)	0.001	5.162 (1.888–14.109)	0.001	2.870 (1.358–6.063)	0.006	2.499 (1.020–6.123)	0.045

## Discussion

It is reasonable to assume that there would be more CTCs in patients with HCC and PVTT due to the greater accessibility of tumor cells in the thrombus to the blood stream. An association between CTC and vascular invasion or PVTT has been shown in data from several previous studies (Sun 2013; Fan 2015; Ogle 2016; Zhou 2016). However, the role of CTCs in HCC with PVTT is not fully understood. Therefore, this retrospective study was designed to investigate the role of CTC in HCC patients with PVTT. Previous studies have shown that both PVTT and CTC are strongly associated with HCC progression (Chen 2006; Schulze 2013; Sun 2013; Bae 2020), so while we were analyzing the full cohort data, we also conduct propensity score analysis in this study to exclude the interference of these factors.

The main findings of this study are twofold. Firstly, we found a significant association between preoperative CTC counts and presence of PVTT in HCC patients undergoing curative liver resection. This was well illustrated by the results of the propensity score matching analysis when other clinical factors were excluded. HCC patients with PVTT were more probable to have higher preoperative CTC counts than those without. And there is a tendency for the number of CTCs to rise as the level of PVTT invasion rises. Unfortunately, as all patients with PVTT enrolled in this study underwent hepatectomy and had type I-II PVTT, we lack further data on high-grade PVTT type. Secondly, we confirmed the prognostic value of preoperative CTC in HCC patients who underwent curative liver resection, especially in those with PVTT. We found that preoperative CTC demonstrated significant prognostic value both in the whole cohort, in the PSM model, and in HCC patients with PVTT; patients with preoperative CTC ≥ 2 had poorer long-term OS and DFS compared to those with preoperative CTC < 2.

There were some limitations in this study. First, this was a retrospective single-center analysis, and the number of HCC patients with PVTT included in this investigation was limited. This caused the role of individual outliers to be magnified in the statistics, which was reflected in the results. Although the prognostic value of CTC was highly significant, some other factors that have been reported to be associated with HCC prognosis, such as tumor size and pathological grade, were shown to be insignificant in some statistical analysis of this study. However, the primary goal of this study is to examine the utility of CTC as a biomarker in HCC patients with PVTT who are receiving surgical treatment. Clinically, fewer of such patients are amenable to surgical intervention, and the comparison of prognostic differences among patients receiving various forms of treatment would lose much of its significance. As a result, we decided on the study’s inclusion criteria.

Secondly, the CellSearch technology was utilized to detect CTC counts in HCC patients. Despite the increasing reports of CTC detection in HCC utilizing this approach, its application in HCC is still regarded inadequate since its detection rate of CTC appears to be associated with EpCAM expression in individual tumors (Went 2004). Only approximately 35% of HCC cases express EpCAM (Yamashita 2008), so there would be predictable low detection sensitivity and a large number of false negative results.

## Conclusion

In this study, we investigated the role of preoperative circulating tumor cells (CTC) in hepatocellular carcinoma (HCC) patients with portal vein tumor thrombosis (PVTT) who underwent curative liver resection. We found a link between preoperative CTC counts and the occurrence of PVTT and confirmed the prognostic significance of preoperative CTC counts in HCC patients with PVTT. These findings suggest that preoperative CTC have the potential to assist in identifying patients with HCC and PVTT who may benefit from surgical therapy. Future research should focus on developing more sensitive CTC detection systems for the characteristics of HCC and PVTT, leading to multicenter prospective studies to explore the value and protocols for clinical application of CTC in these patients.

## Supplementary Information

Below is the link to the electronic supplementary material.

Supplementary file1 Comparison of preoperative CTC counts in HCC patients without MVI versus HCC patients with MVI in the full cohort, Mann-Whitney test, P < 0.0001 (TIF 895 KB)

## Data Availability

The datasets generated during and/or analyzed during the current study are available from the corresponding author on reasonable request.
