# Supplementary figures and images for: Prognostic value of preoperative circulating tumor cells for hepatocellular carcinoma with portal vein tumor thrombosis: A propensity score analysis
Source: J Cancer Res Clin Oncol. 2023 May 9;149(11):8981–91. doi: 10.1007/s00432-023-04834-8 (PMC10374672; doi:10.1007/s00432-023-04834-8)

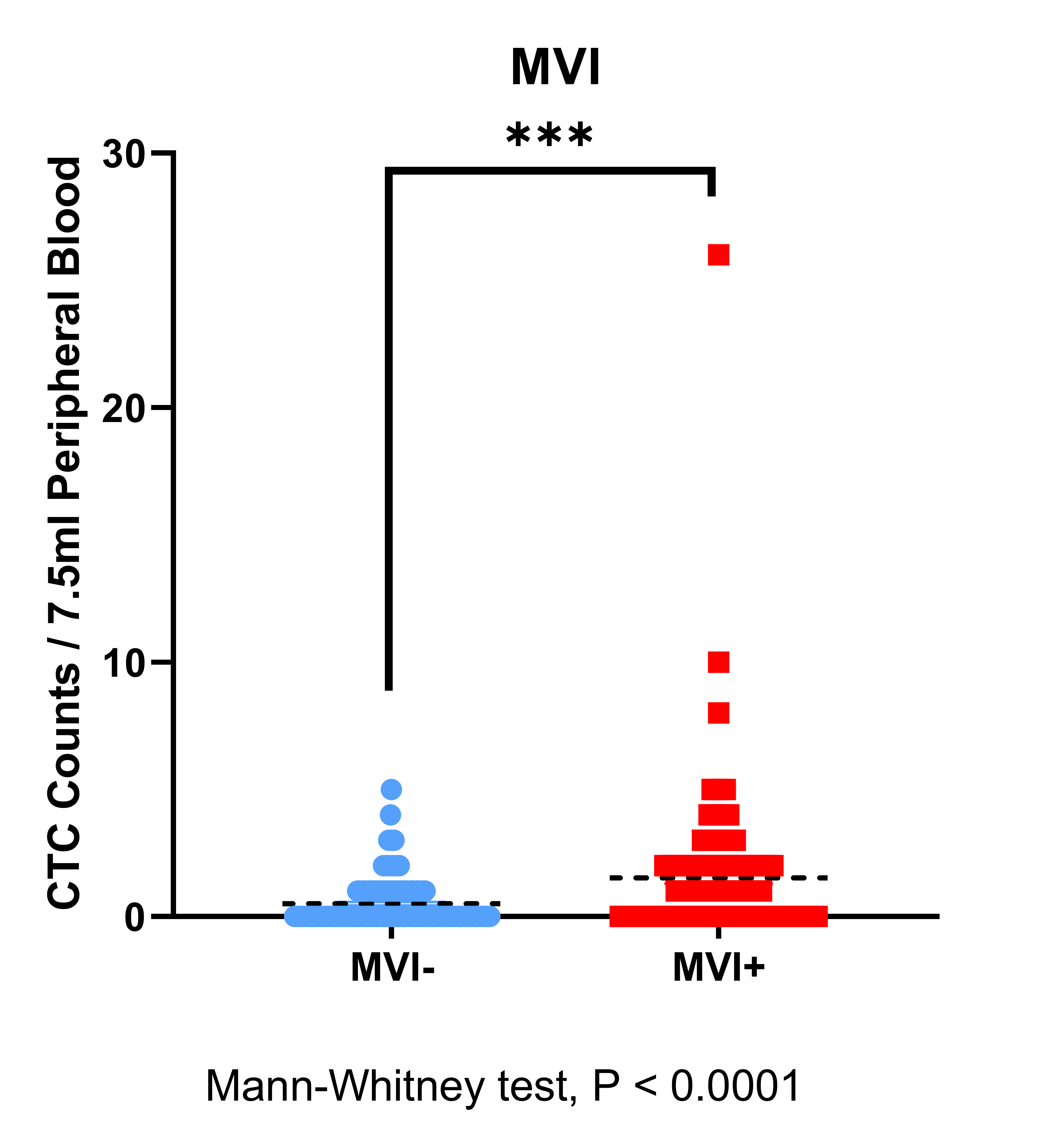

Supplement: Supplementary file 1 — Supplementary file1 Comparison of preoperative CTC counts in HCC patients without MVI versus HCC patients with MVI in the full cohort, Mann-Whitney test, P < 0.0001 (TIF 895 KB) [file 432_2023_4834_MOESM1_ESM.tif]
